# Multimodal Non-Surgical Management for Chronic Low Back Pain: A 5-Year Cohort Study from a Tertiary Spine Center in Northwest China

**DOI:** 10.3390/jcm15166497

**Published:** 2026-08-21

**Authors:** Bolong Zheng, Hua Hui, Liang Yan, Baorong He

**Affiliations:** Department of Spine Surgery, Honghui Hospital, Xi’an Jiaotong University, Xi’an 710054, Shaanxi, China; jason_spine@163.com (B.Z.);

**Keywords:** chronic low back pain, multimodal non-surgical management, multidisciplinary biopsychosocial care, pain catastrophizing, long-term outcomes

## Abstract

**Background/Objectives:** Chronic low back pain (CLBP) is the leading global cause of years lived with disability, but long-term real-world evidence for non-surgical management remains scarce, particularly in low- and middle-income countries. This study aimed to describe 5-year trajectories of pain, disability, healthcare utilization, patient satisfaction, and surgical conversion among patients with non-specific CLBP initially managed non-surgically at a tertiary spine center in Northwest China. We also compared long-term outcomes between unimodal therapy and multidisciplinary biopsychosocial care and examined baseline demographic, clinical, and psychosocial predictors of treatment success and conversion to surgery. **Methods:** We conducted a 5-year retrospective cohort study of consecutive patients with non-specific CLBP at a tertiary spine center in Northwest China. Of 485 consecutive patients screened for eligibility, 420 were enrolled and were compared on the basis of unimodal therapy with multidisciplinary biopsychosocial care. Primary outcomes were pain (Numerical Pain Rating Scale, NPRS) and disability (Oswestry Disability Index, ODI), analyzed using linear mixed-effects models; predictors of surgical conversion were identified via multivariable Cox regression. **Results:** Of the 420 patients, 352 (83.8% retention) completed the 5-year follow-up. Both pain and disability improved substantially during the first 12 months. Thereafter, pain intensity increased slightly (NPRS from 4.8 to 5.4), whereas functional disability continued to improve (ODI from 31.7 to 26.5). Early multidisciplinary care was associated with sustained superior outcomes (adjusted functional disability (ODI) difference: −8.2 points at 5 years) and a 42% lower observed risk of surgery (HR = 0.58). The cumulative 5-year surgical rate was 14.2%, with high pain catastrophizing as the strongest independent predictor (HR = 3.10). **Conclusions:** In patients with non-specific CLBP, non-surgical management yields substantial 12-month gains; function continues to improve through 5 years, while pain shows partial recurrence. Early multidisciplinary biopsychosocial care was associated with more durable outcomes and lower surgical conversion; because treatment was not randomized, these associations warrant confirmation in pragmatic trials and support routine psychosocial screening and stratified care.

## 1. Introduction

Chronic low back pain (CLBP), usually defined as pain between the lower rib margin and gluteal folds that persists for 12 weeks or longer, is one of the most burdensome musculoskeletal problems worldwide. A Global Burden of Disease analysis estimated that 619 million people were affected by low back pain in 2020 and projected 843 million prevalent cases by 2050, confirming its position as the leading cause of years lived with disability globally [1]. In China, GBD 2021-based estimates show that prevalent low back pain cases increased from 69.61 million in 1990 to 102.96 million in 2021 [2]. A China-specific analysis of GBD 2019 data further identified occupational ergonomic exposure, smoking, and high body mass index as major contributors to the national disability burden, emphasizing the need for prevention and long-term management strategies tailored to the Chinese healthcare context [3].

CLBP is not a homogeneous structural disorder but a symptom complex shaped by nociceptive, neurological, psychological, lifestyle, and social contributors [4]. Approximately 90% of low back pain is classified as non-specific because no single disease or structural lesion can fully explain the symptoms [5]. Degenerative changes on spine imaging are common in asymptomatic adults and increase with age, limiting the value of imaging findings when interpreted without the clinical context [6]. Consequently, for non-specific CLBP, contemporary management prioritizes reduction in pain-related disability, restoration of function, and self-management rather than pursuit of a single anatomical pain generator [7].

Theoretical and clinical frameworks have evolved accordingly. Engel’s biopsychosocial model challenged a disease-only biomedical approach and emphasized interactions among biological processes, psychological responses, and social context [8]. Waddell subsequently adapted this model to low back pain and highlighted how fear, distress, illness behavior, and work or family context can amplify disability even when tissue pathology is not progressive [9]. Current guidelines therefore recommend non-surgical, person-centered care as the first-line strategy for chronic primary or non-specific low back pain. The WHO guideline recommends education, exercise, selected physical therapies, psychological interventions, and appropriate medicines in primary and community care [10], while the ACP guideline similarly prioritizes nonpharmacologic therapies and reserves medication for situations in which these approaches are insufficient [11]. The 2018 Lancet Low Back Pain Series also called for reduced use of low-value imaging, opioids, and surgery and greater investment in active, multidisciplinary care pathways [12].

Among non-surgical options, exercise therapy has the strongest evidence base; a Cochrane review found moderate-certainty evidence that exercise is probably more effective than no treatment/usual care for pain in adults with chronic low back pain [13]. Multidisciplinary biopsychosocial rehabilitation integrates physical reconditioning with psychological and social components and has shown benefits over usual care or isolated physical treatments, especially for disability [14]. Pain neuroscience education may further support recovery by reconceptualizing pain, reducing threat appraisal, and improving engagement with movement when combined with physiotherapy or exercise [15]. Nevertheless, durable long-term effects remain uncertain. A 2025 systematic review and meta-analysis of 75 trials involving 15,395 participants concluded that cognitive behavioral therapy, mindfulness, exercise, and multidisciplinary care may provide long-term benefits, but effect sizes were generally small and evidence certainty varied [16].

This uncertainty is clinically important because low back pain often follows recurrent or persistent trajectories rather than a simple episode-recovery pattern [17]. Five-year trajectory research has shown that baseline pain, disability, and psychosocial status can help identify patients likely to remain in unfavorable long-term courses [18]. Among psychosocial factors, pain catastrophizing has been identified as a prognostic factor for greater pain and disability, supporting the use of early psychological screening in CLBP care [19]. Stratified care models such as STarT Back demonstrate that matching treatment intensity to prognostic risk can improve outcomes and resource use compared with non-stratified current best practice [20].

Despite these advances, several evidence gaps remain. Most trials test standardized interventions under controlled conditions and provide limited information on how multimodal care performs over 5 years in routine tertiary spine practice. Data from Chinese cohorts are particularly limited, although China has a large and aging population, rapidly changing occupational exposures, and regional differences in access to rehabilitation and psychological services. Real-world cohort studies can complement randomized trials by capturing clinical heterogeneity, patient adherence, treatment modification, and surgical conversion over extended follow-up.

Therefore, this study aimed to describe 5-year trajectories of pain, disability, healthcare utilization, patient satisfaction, and surgical conversion among patients with non-specific CLBP initially managed non-surgically at a tertiary spine center in Northwest China. We also compared long-term outcomes between unimodal therapy and multidisciplinary biopsychosocial care and examined baseline demographic, clinical, and psychosocial predictors of treatment success and conversion to surgery. We hypothesized that non-surgical care would produce meaningful short-term improvements, with partial recurrence of pain over time while function was maintained or continued to improve, that multidisciplinary care would be associated with more durable outcomes and lower surgical conversion rates than unimodal therapy, and that psychosocial risk factors would be stronger predictors of long-term outcomes than baseline physical impairment alone.

## 2. Materials and Methods

### 2.1. Study Design and Ethical Approval

This was a single-center, retrospective study of patient materials conducted at the Department of Spine Surgery, Xi’an Honghui Hospital, a 3000-bed tertiary academic medical center specializing in orthopedic and spinal disorders. The study was approved by the Institutional Review Board (IRB) of Xi’an Honghui Hospital (Approval No. 2026-KY-127-01; approved 5 June 2026). The original clinical care delivered during the 2019–2020 enrollment period was provided under routine clinical consent; the June 2026 IRB approval, which included a waiver of written informed consent for research use of de-identified data, governed the retrospective EMR analysis and the January–February 2026 follow-up interviews, and no research procedures were performed before IRB approval. Due to the retrospective nature of the study and the use of de-identified electronic medical record (EMR) data, the IRB waived the requirement for written informed consent.

### 2.2. Study Population

We screened all consecutive patients aged ≥18 years who presented to the spine surgery outpatient clinic with a primary diagnosis of non-specific CLBP between 1 January 2019 and 31 December 2020. The index date was defined as the date of the first consultation during this 2-year enrollment period. Non-specific CLBP was defined as pain localized between the lower costal margin and the gluteal folds, with or without referred pain to the posterior thigh (above the knee), lasting for ≥12 consecutive weeks, and without a specific underlying pathoanatomical diagnosis (e.g., tumor, infection, inflammatory spondyloarthropathy, acute fracture, or cauda equina syndrome) on clinical assessment and available imaging. Patients with distinct radicular pain radiating below the knee in a dermatomal distribution with corresponding neurological signs (motor weakness, reflex change, or dermatomal sensory loss) were classified as having radiculopathy/sciatica rather than non-specific CLBP and were excluded. Referred thigh pain was permitted, which accounts for the 31% of patients who reported “leg pain” at baseline (Table 1); this represented somatic referred pain rather than true radiculopathy. During follow-up, surgery was considered in patients who developed progressive or new radiculopathy, symptomatic spinal stenosis with neurogenic claudication, or segmental instability confirmed on imaging, or whose symptoms remained refractory to exhaustive non-surgical care; the decision was made jointly by the treating spine surgeon and patient and was not determined by the study protocol.

Inclusion criteria: Age ≥18 years at index visit; diagnosis of non-specific CLBP meeting the above definition; initial management plan documented in the EMR consisting exclusively of non-surgical interventions; availability of complete baseline outcome measures (NPRS and ODI); resident of Shaanxi province or adjacent regions to ensure feasibility of long-term follow-up.

Exclusion criteria: History of any spinal surgery prior to the index date; specific spinal pathologies including malignancy, spinal infection, ankylosing spondylitis, rheumatoid arthritis affecting the spine, symptomatic spondylolisthesis > grade II, cauda equina syndrome, spinal fracture, or spinal stenosis with progressive neurological deficits; major systemic comorbidities that would independently impair mobility or function, including severe neuromuscular disease, end-stage renal disease, congestive heart failure (NYHA class III-IV), and active malignancy; pregnancy or lactation at index visit; incomplete baseline demographic or clinical data; participation in another clinical trial involving CLBP treatment during the study period.

### 2.3. Data Sources and Collection

Baseline data were extracted from the hospital’s Wellbeing EMR v4.1 system, which includes comprehensive information on patient demographics, medical history, physical examination findings, imaging results, treatment plans, and outpatient visit records. Data extraction was performed by two trained research assistants using a standardized data collection form, with 10% of records independently reviewed by a senior researcher to ensure accuracy (inter-rater agreement = 0.96).

Follow-up data for the first 2 years (3, 6, and 12 months) were obtained from routine outpatient clinic visits, where outcome measures were administered by attending physicians or clinic nurses as part of standard clinical care. Two-year (24-month) data were likewise collected during scheduled outpatient review. Because resource constraints precluded separate annual research visits in years 3, 4, and 5, the 36-, 48-, and 60-month assessments were obtained during a single structured telephone-interview window between 5 June and 4 July 2026. During this call, trained research nurses administered the NPRS, ODI, TSK-11, PCS, and secondary-outcome items for each of the three annual time points, prompting patients to report their status at approximately 3, 4, and 5 years after enrolment with the aid of anchored recall questions (e.g., “Thinking back to around [month/year], how severe was your back pain on average?”). The interview script was developed based on validated questionnaires and pilot-tested on 20 patients to ensure clarity and comprehensibility. Interviews were conducted by three research nurses who received 8 h of specialized training in administering the outcome measures and conducting standardized interviews. Inter-rater reliability was assessed prior to data collection, with intraclass correlation coefficients (ICCs) of 0.92 for NPRS and 0.89 for ODI, indicating excellent reliability. We acknowledge that simultaneous retrospective collection of the 3- and 4-year data, in addition to the 5-year data, introduces the possibility of differential recall across time points; this is addressed as a limitation in Section 4.5.

### 2.4. Intervention Classification

Patients received non-surgical management at the discretion of their attending spine surgeon, in accordance with contemporary clinical guidelines. Based on the initial treatment plan documented in the EMR, patients were classified into two distinct treatment groups:

Unimodal Therapy Group (n = 234): Patients received management consisting primarily of a single intervention modality, either: (1) Pharmacological treatment, including non-steroidal anti-inflammatory drugs (NSAIDs), muscle relaxants, and acetaminophen. Medication prescriptions were individualized based on patient symptoms and comorbidities, with typical treatment durations ranging from 2 to 12 weeks. (2) Single-modality physical therapy, including basic stretching and strengthening exercises, traction, electrotherapy (TENS, ultrasound), or manual therapy. Physical therapy was typically prescribed 2–3 times per week for 4–6 weeks, with home exercise programs provided to patients upon completion.

Multidisciplinary Care Group (n = 118): Patients received a structured, 12-week multidisciplinary program delivered by an interdisciplinary team consisting of a spine surgeon, physical therapist, clinical psychologist, and occupational therapist. The program included four core components, delivered over 12 weeks and comprising 24 supervised exercise sessions, 4 pain-neuroscience education group sessions, and 2 individual occupational-therapy sessions, with cognitive-behavioral principles integrated throughout: (1) Supervised, tailored exercise therapy: 1 h sessions twice weekly (24 sessions over 12 weeks), focusing on core stabilization, motor control, and functional restoration, with exercises progressed based on individual patient tolerance and goals; (2) pain neuroscience education: four 60 min group sessions delivered by the clinical psychologist, covering the neurobiology of pain, the role of central sensitization, and reconceptualization of pain as a marker of tissue damage; (3) cognitive-behavioral-based physical therapy integrating graded activity, exposure to feared movements, and goal-setting, delivered jointly by the physical therapist and psychologist; (4) occupational therapy: two 45 min individual sessions delivered by the occupational therapist covering workplace ergonomic assessment, activity-pacing strategies, body-mechanics retraining, and graded return-to-activity and work-planning, with ergonomic and pacing principles also reinforced during the supervised exercise and cognitive-behavioral sessions to support transfer of rehabilitation gains to daily work and home routines.

Adherence to the multidisciplinary protocol was defined as attending at least 16 of the 24 supervised exercise sessions (i.e., ≥67%) and at least 3 of the 4 pain-neuroscience education sessions. Patients who did not meet this adherence criterion were still included in the multidisciplinary group for intention-to-treat analysis. The mean number of exercise sessions attended was 19.6 ± 4.2 (median 21; 81.7% of patients met the adherence threshold), and the mean number of education sessions attended was 3.5 ± 0.8. During the 5-year follow-up, 28.0% of patients in the multidisciplinary group and 41.9% in the unimodal group received at least one additional course of physical therapy outside the index program; these subsequent treatments were not controlled and are described in Section 3.5.

### 2.5. Outcome Measures

Primary Outcomes: (1) Pain Intensity: Measured using the 11-point Numerical Pain Rating Scale (NPRS), where 0 = no pain and 10 = worst imaginable pain. Patients were asked to rate their average pain intensity over the past 7 days. (2) Functional Disability: Measured using the Chinese version of the Oswestry Disability Index (ODI) version 2.0, a 10-item questionnaire assessing disability in activities of daily living, including personal care, lifting, walking, sitting, standing, sleeping, sex life, social life, and travel. Scores range from 0 to 100, with higher scores indicating greater disability. The Chinese version of the ODI has been validated in Chinese populations with CLBP, demonstrating excellent internal consistency (Cronbach’s alpha = 0.89) and test–retest reliability (ICC = 0.92).

Primary outcomes were assessed at baseline, 3 months, 6 months, 12 months, 2 years, 3 years, 4 years, and 5 years.

Secondary Outcomes: (1) Surgical Conversion: Incidence of any lumbar spine surgery (discectomy, laminectomy, fusion, or disc replacement) during the 5-year follow-up period, confirmed by review of hospital surgical records. (2) Healthcare Utilization: Number of outpatient visits to the spine surgery clinic, number of emergency department visits for back pain, and number of hospitalizations for back pain in the 12 months prior to the 5-year follow-up. (3) Medication Usage: Average number of days per month that patients used analgesic medications (NSAIDs, muscle relaxants, opioids) for back pain in the past 3 months. (4) Patient Satisfaction: Assessed at 5 years using a 5-point Likert scale (1 = very dissatisfied, 2 = dissatisfied, 3 = neutral, 4 = satisfied, 5 = very satisfied). (5) Global Perceived Effect (GPE): Assessed at 5 years using a 7-point scale asking patients to rate their current condition compared to before treatment (1 = very much worse, 2 = much worse, 3 = slightly worse, 4 = unchanged, 5 = slightly better, 6 = much better, 7 = very much better). For analysis, responses were dichotomized as “improved” (scores 5–7) and “not improved” (scores 1–4).

### 2.6. Covariates

We collected data on potential confounding variables that have been shown to influence CLBP outcomes in previous studies: (1) Demographics: Age (years), sex (male/female), body mass index (BMI, kg/m^2^), marital status, education level, and employment status. (2) Baseline Clinical Characteristics: Duration of current pain episode (months), presence of leg pain/sciatica (yes/no), baseline NPRS and ODI scores, number of prior non-surgical treatments for back pain, and smoking status (current smoker/former smoker/never smoker). (3) Psychosocial Factors: Baseline scores on the 11-item Tampa Scale for Kinesiophobia (TSK-11), which measures fear of movement/re-injury (scores range from 11 to 44, with higher scores indicating greater kinesiophobia), and the 13-item Pain Catastrophizing Scale (PCS), which measures the tendency to magnify pain threat, ruminate on pain, and feel helpless in the face of pain (scores range from 0 to 52, with higher scores indicating greater catastrophizing). (4) Comorbidities: Assessed using the Charlson Comorbidity Index (CCI), a weighted index that predicts 10-year mortality based on the presence of 19 comorbid conditions.

### 2.7. Statistical Analysis

Sample size was calculated based on the primary outcome of ODI score at 5 years. Assuming a clinically meaningful difference in ODI of 8 points between the multidisciplinary and unimodal groups, a standard deviation of 10 points, an alpha level of 0.05, and a power of 0.8, we estimated that a total of 100 patients per group would be required. Accounting for an estimated 20% loss to follow-up, we aimed to enroll 250 patients in total. However, due to the high volume of patients at our center, we were able to enroll 420 consecutive patients, providing greater statistical power to detect smaller differences and perform subgroup analyses.

Descriptive statistics were calculated for all baseline variables. Continuous variables are presented as mean ± standard deviation (SD) for normally distributed data or median (interquartile range [IQR]) for non-normally distributed data. Categorical variables are presented as frequencies and percentages. Differences in baseline characteristics between the unimodal and multidisciplinary groups were compared using independent samples t-tests for continuous variables and chi-square tests for categorical variables.

To analyze longitudinal changes in NPRS and ODI over the 5-year follow-up period, we used linear mixed-effects models (LMMs) with random intercepts and random slopes for individuals. This approach accounts for the correlation between repeated measures within the same patient and allows for inclusion of patients with missing data at some time points. The primary longitudinal analyses included all 420 enrolled patients under a missing-at-random assumption, with missing follow-up values handled by multiple imputation by chained equations (MICE; see below); complete-case data (n = 352) were used for descriptive tables and as a sensitivity analysis. Time was modeled as a categorical variable (months since baseline) to capture non-linear changes in outcomes over time. We built three nested models for each outcome: (1) an unconditional growth model with time only; (2) a model adjusting for demographic variables (age, sex, BMI); and (3) a fully adjusted model additionally including clinical and psychosocial covariates (duration of pain, leg pain, smoking status, TSK-11, PCS, CCI, and treatment group). Model fit was compared using likelihood-ratio tests, with the fully adjusted model retained as the primary inferential model.

To compare outcomes between the unimodal and multidisciplinary groups, we included an interaction term between treatment group and time in the LMMs. Adjusted mean differences and 95% confidence intervals (CIs) were calculated for each time point. Subgroup analyses were performed to assess whether the effect of multidisciplinary care varied by baseline psychosocial risk (high vs. low, defined by TSK-11 > 35 or PCS > 24), age (<50 vs. ≥50 years), sex, and duration of pain (<1 year vs. ≥1 year). Interaction terms were included in the models to test for statistically significant subgroup effects.

Predictors of surgical conversion were analyzed using multivariable Cox proportional-hazards models. With 50 observed surgical events, the multivariable model was constrained a priori to five covariates to maintain an events-per-variable (EPV) ratio of ≥10 (50/5 = 10), in line with standard prognostic-modelling guidance. The five covariates were selected on clinical grounds from among variables with *p* < 0.20 in univariable analysis: baseline ODI, leg pain, high PCS (>24), current smoking, and treatment group. Variables with a *p*-value < 0.20 in univariable analysis but not retained in the parsimonious model (age, sex, BMI, duration of pain, TSK-11, CCI) are reported in Table 2 for transparency. The proportional-hazards assumption was tested using Schoenfeld residuals, and no violations were detected. Given that 50 events is at the lower threshold for stable multivariable estimation, we additionally present Firth’s penalized-likelihood Cox model (coxphf in R) as a complementary primary analysis to reduce small-sample and sparse-data bias. Point estimates and 95% CIs from the penalized model were virtually identical to those of the standard Cox model (e.g., PCS >24: HR 3.04, 95% CI 1.76–5.25; multidisciplinary care: HR 0.60, 95% CI 0.39–0.91), and we report the penalized estimates alongside the standard model throughout. Kaplan–Meier survival curves were generated to illustrate cumulative surgical rates over time, and differences between groups were tested using log-rank tests.

Missing data were handled using multiple imputation by chained equations (MICE), assuming data were missing at random. Twenty imputed datasets were generated, and results were pooled using Rubin’s rules. Sensitivity analyses were performed using complete-case analysis (excluding patients with any missing outcome data) and alternative model specifications to confirm the robustness of our findings.

All statistical tests were two-sided, with a significance level set at *p* < 0.05. Analyses were performed using R software version 4.3.0 (R Foundation for Statistical Computing, Vienna, Austria) using the lme4, mice, survival, and ggplot2 packages.

## 3. Results

### 3.1. Patient Flow and Baseline Characteristics

A total of 485 consecutive patients with non-specific CLBP were screened for eligibility between 1 January 2019 and 31 December 2020. Of these, 65 patients were excluded: 28 had prior spinal surgery, 15 had specific spinal pathologies, 12 had major systemic comorbidities, and 10 had incomplete baseline data. The remaining 420 patients were included in the initial cohort. Follow-up rates at each scheduled assessment were: 94.0% (n = 395) at 3 months, 91.7% (n = 385) at 6 months, 89.0% (n = 374) at 12 months, 86.9% (n = 365) at 24 months, 85.7% (n = 360) at 36 months, 84.8% (n = 356) at 48 months, and 83.8% (n = 352) at 60 months. At the 5-year follow-up, 352 patients (83.8%) had complete outcome data and were included in the final analysis. Reasons for loss to follow-up among the 68 patients not assessed at 5 years included inability to contact (n = 37), withdrawal of consent (n = 18), relocation outside the region (n = 9), and death unrelated to back pain (n = 4). Patients lost to follow-up did not differ significantly from retained patients in age, sex, baseline NPRS, or baseline ODI (all *p* > 0.10), although they were more likely to be current smokers (22.1% vs. 13.4%, *p* = 0.047).

Baseline characteristics of the final study cohort are presented in Table 1. The mean age was 49.2 ± 11.8 years (range: 19–78 years), and 199 patients (56.5%) were female. The mean BMI was 25.1 ± 3.8 kg/m^2^, with 32.4% of patients classified as overweight (BMI 25–29.9 kg/m^2^) and 12.8% classified as obese (BMI ≥30 kg/m^2^). The median duration of pain prior to the index visit was 18 months (IQR: 6–48 months), with 65.6% of patients reporting pain lasting >1 year. At baseline, patients exhibited moderate-to-severe pain (mean NPRS = 7.1 ± 1.4) and disability (mean ODI = 44.8 ± 9.7), with 78.4% of patients having ODI scores ≥40, indicating severe disability. Psychosocial risk factors were highly prevalent: the mean TSK-11 score was 33.1 ± 6.5, and the mean PCS score was 25.2 ± 7.9, with 52.3% of patients having PCS scores >24, indicating clinically significant pain catastrophizing. Ninety-five patients (27.0%) were current smokers, and the mean CCI score was 1.2 ± 1.5, indicating a low overall burden of comorbidities.

Of the 352 patients, 234 (66.5%) were initially managed with unimodal therapy, while 118 (33.5%) received multidisciplinary care. Comparison of baseline characteristics between the two groups revealed several statistically significant differences (Table 1). Patients in the multidisciplinary group were younger (47.5 ± 12.1 vs. 50.1 ± 11.5 years, *p* = 0.04), had higher baseline ODI scores (47.4 ± 10.1 vs. 43.5 ± 9.2, *p* < 0.001), and had significantly higher psychosocial risk scores (mean TSK-11: 35.8 ± 6.4 vs. 31.8 ± 6.2, *p* < 0.001; mean PCS: 28.6 ± 7.8 vs. 23.5 ± 7.6, *p* < 0.001). There were no significant differences between groups in sex, BMI, duration of pain, baseline NPRS, smoking status, or CCI score. These differences were adjusted for in all subsequent multivariable analyses.

### 3.2. Longitudinal Trajectories of Pain and Disability

**Figure 1:**
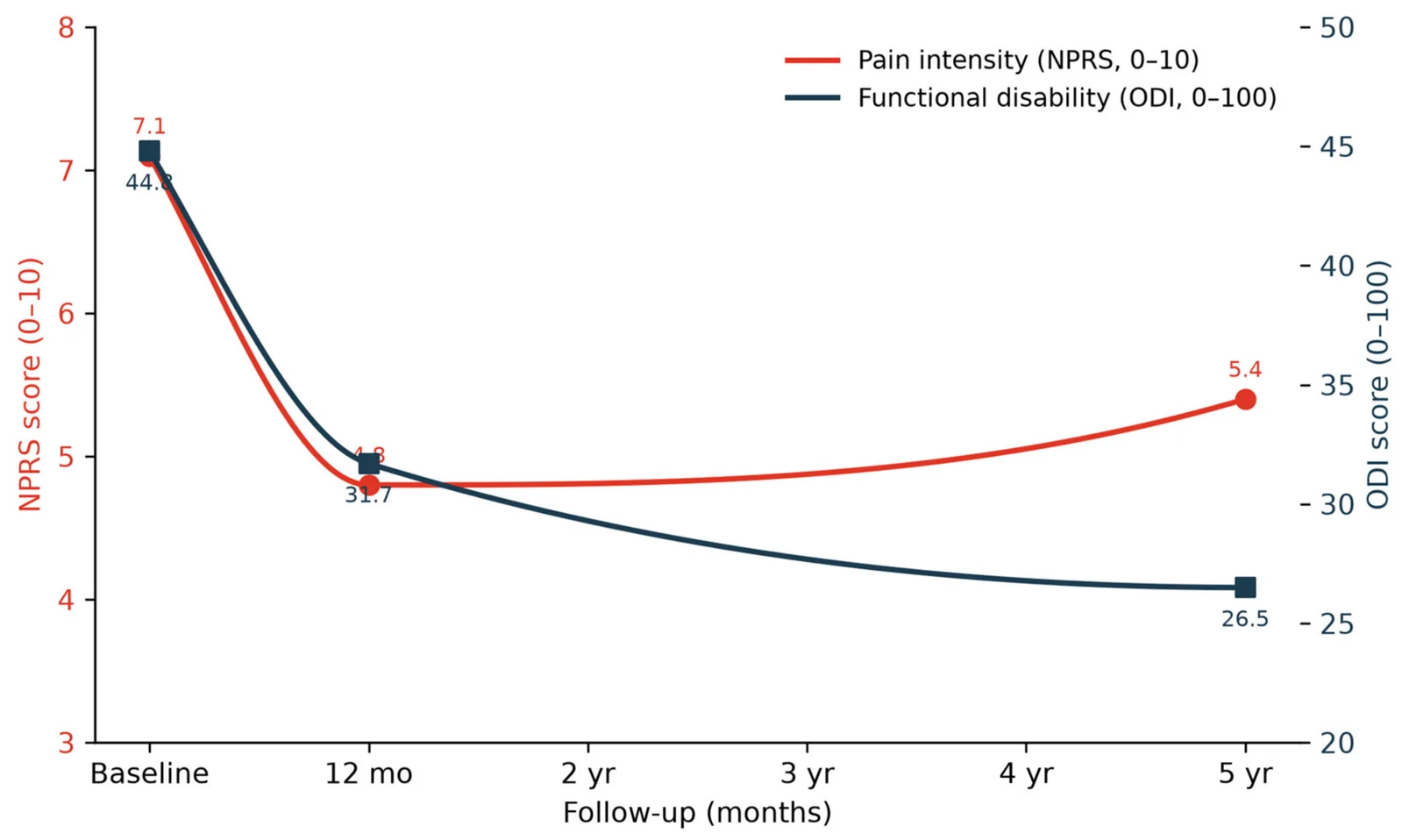
Five-year trajectories of pain intensity (Numerical Pain Rating Scale, NPRS) and functional disability (Oswestry Disability Index, ODI) for the entire cohort (n = 352). Values are observed means at baseline, 12 months, and 5 years; lines are monotone interpolations for visual trend. Both outcomes improved during the first year; pain intensity rose slightly thereafter (4.8 → 5.4), while disability continued to improve gradually (31.7 → 26.5).

Figure 1 illustrates the mean trajectories of pain intensity (NPRS) and functional disability (ODI) over the 5-year follow-up period for the entire cohort. Both pain and disability improved rapidly during the first 12 months of treatment. The mean pain intensity (NPRS) decreased from 7.1 ± 1.4 at baseline to 4.8 ± 1.7 at 12 months, representing a 32.4% reduction in pain intensity (adjusted mean difference: −2.3 points, 95% CI: −2.5 to −2.1, *p* < 0.001). The mean functional disability (ODI) decreased from 44.8 ± 9.7 at baseline to 31.7 ± 11.2 at 12 months, representing a 29.2% reduction in disability (adjusted mean difference: −13.1 points, 95% CI: −14.2 to −12.0, *p* < 0.001).

After reaching a nadir at 12 months, the two primary outcomes diverged over the subsequent 4 years. From 12 months to 5 years, mean pain intensity (NPRS) increased by 0.6 points (95% CI: 0.4 to 0.8, *p* < 0.001) to 5.4 ± 1.9, indicating partial recurrence of pain, whereas mean functional disability (ODI) decreased by a further 5.2 points (95% CI: −6.5 to −3.9, *p* < 0.001) to 26.5 ± 10.8, indicating continued functional improvement. Despite the slight recurrence of pain, both outcomes remained significantly improved from baseline at 5 years (pain intensity (NPRS): adjusted mean difference −1.7 points, 95% CI: −1.9 to −1.5, *p* < 0.001; functional disability (ODI): adjusted mean difference −18.3 points, 95% CI: −19.7 to −16.9, *p* < 0.001).

The linear mixed-effects model confirmed that time was a highly significant predictor of both pain intensity (NPRS) and functional disability (ODI) (*p* < 0.001 for all models). Adjustment for demographic, clinical, and psychosocial variables did not substantially alter the trajectory of outcomes, indicating that the observed pattern—rapid early improvement in both outcomes followed by slight pain recurrence and sustained functional gain—was consistent across patient subgroups.

### 3.3. Comparison of Unimodal Versus Multidisciplinary Care

**Figure 2:**
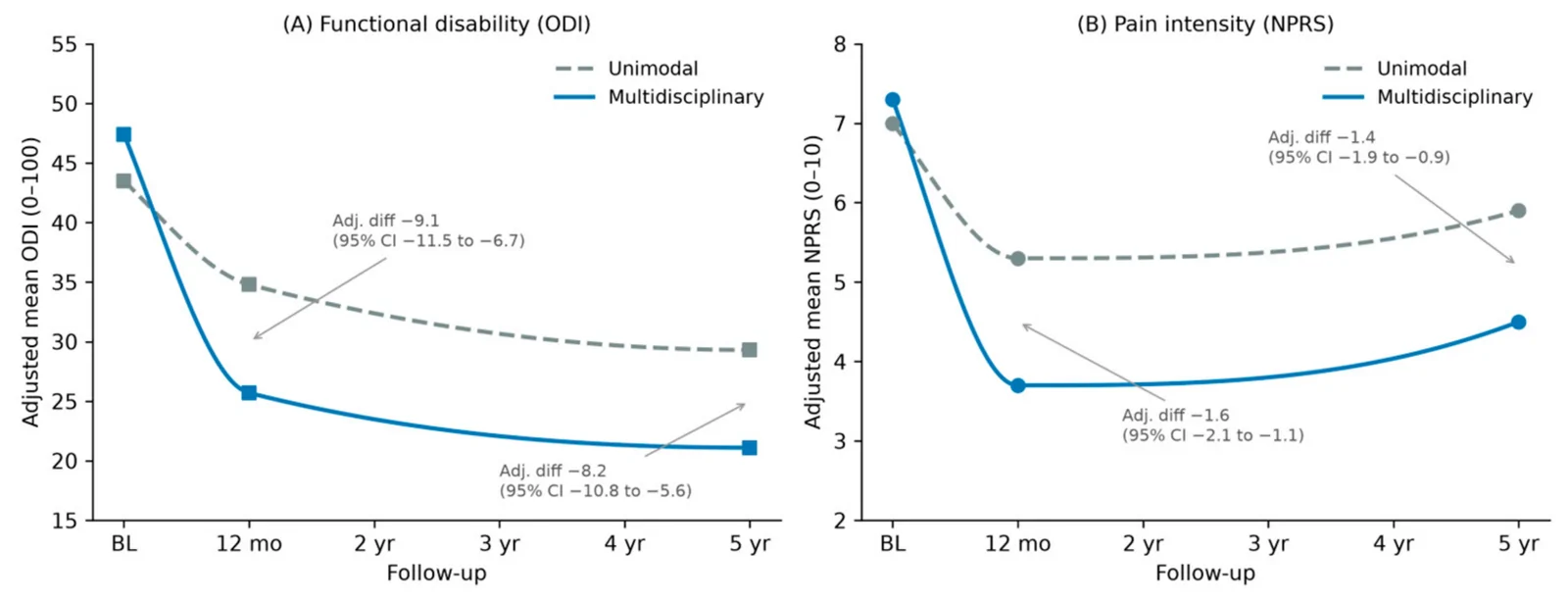
Adjusted mean (**A**) functional disability (ODI) and (**B**) pain intensity (NPRS) over 5 years, by treatment group. Values are adjusted means from linear mixed-effects models at baseline, 12 months, and 5 years; lines are monotone interpolations. The multidisciplinary group showed significantly lower disability and pain than the unimodal group at all post-baseline time points, with adjusted between-group differences of −8.2 ODI points and −1.4 NPRS points at 5 years.

Patients receiving multidisciplinary care had significantly better outcomes in both pain and disability across all follow-up time points compared to patients receiving unimodal therapy (Figure 2). At 12 months, the adjusted mean difference in functional disability (ODI) between the multidisciplinary and unimodal groups was −9.1 points (95% CI: −11.5 to −6.7, *p* < 0.001), and the adjusted mean difference in pain intensity (NPRS) was −1.6 points (95% CI: −2.1 to −1.1, *p* < 0.001). This advantage persisted throughout the 5-year follow-up period, with an adjusted mean difference in functional disability (ODI) of −8.2 points (95% CI: −10.8 to −5.6, *p* < 0.001) and in pain intensity (NPRS) of −1.4 points (95% CI: −1.9 to −0.9, *p* < 0.001) at 5 years.

Subgroup analysis revealed that the benefit of multidisciplinary care was most pronounced in patients with high baseline psychosocial risk (fear of movement as measured by the 11-item Tampa Scale for Kinesiophobia (TSK-11) > 35 or pain catastrophizing as measured by the Pain Catastrophizing Scale (PCS) > 24). In this high-risk subgroup (n = 184), the 5-year adjusted mean functional disability (ODI) was 22.8 ± 9.5 in the multidisciplinary group versus 33.5 ± 10.2 in the unimodal group, representing a difference of 10.7 points (95% CI: −13.9 to −7.5, *p* < 0.001). In contrast, in patients with low baseline psychosocial risk (n = 168), the 5-year adjusted mean functional disability (ODI) difference between groups was only 4.3 points (95% CI: −7.2 to −1.4, *p* = 0.004), which was statistically significant but not clinically meaningful (defined as a difference of ≥8 points). The interaction between treatment group and psychosocial risk was statistically significant (*p* = 0.002), indicating that multidisciplinary care provides a greater benefit in patients with elevated psychosocial risk factors.

### 3.4. Surgical Conversion Rates

Over the 5-year follow-up period, 50 patients (14.2%) underwent lumbar spine surgery. The cumulative incidence of surgery increased steadily over time, with the highest rate of conversion occurring within the first 2 years (9.1% by 24 months), followed by a slower, more gradual increase thereafter (11.6% by 36 months, 13.1% by 48 months, and 14.2% by 60 months). The most common surgical procedures were lumbar discectomy (n = 22, 44.0%), transforaminal lumbar interbody fusion (TLIF) (n = 18, 36.0%), and laminectomy (n = 10, 20.0%).

Kaplan–Meier survival curves demonstrated that patients receiving multidisciplinary care had a significantly lower cumulative surgical conversion rate compared to patients receiving unimodal therapy (log-rank test, *p* = 0.008). At 5 years, the cumulative surgical rate was 8.5% in the multidisciplinary group versus 17.1% in the unimodal group, corresponding to an unadjusted 50% lower absolute surgical rate in the multidisciplinary group; after multivariable adjustment, multidisciplinary care was associated with a 42% lower hazard of surgery (HR 0.58, 95% CI 0.38–0.88). Because treatment allocation was not randomized, this association should be interpreted with caution (see Section 4.2 and Section 4.5).

Results of the univariable and multivariable Cox proportional hazards models for predictors of surgical conversion are presented in Table 2. In univariable analysis, higher baseline functional disability (ODI), presence of leg pain, high pain catastrophizing (PCS) score, current smoking, and unimodal treatment were significantly associated with increased risk of surgical conversion. In the multivariable model, high pain catastrophizing (PCS > 24) remained the strongest independent predictor of surgical conversion (HR 3.10, 95% CI: 1.80–5.34, *p* < 0.001), followed by presence of leg pain/sciatica (HR 2.05, 95% CI: 1.45–2.89, *p* < 0.001), baseline functional disability (ODI) (HR 1.03 per point increase, 95% CI: 1.02–1.04, *p* < 0.001), and current smoking (HR 1.55, 95% CI: 1.10–2.18, *p* = 0.01). Early multidisciplinary care was independently associated with a 42% lower risk of surgical conversion (HR 0.58, 95% CI: 0.38–0.88, *p* = 0.01). Age, sex, BMI, duration of pain, fear of movement (TSK-11) score, and comorbidity burden (CCI) score were not significant predictors of surgical conversion in the multivariable model.

### 3.5. Secondary Outcomes and Patient-Reported Outcomes

Five-year secondary outcomes and patient-reported outcomes are presented in Table 3. Overall, patients reported using analgesic medications for a mean of 7.8 ± 4.5 days per month, with 22.4% of patients using analgesics for ≥15 days per month. The mean number of outpatient visits to the spine surgery clinic in the past year was 3.2 ± 1.8, with 15.3% of patients having ≥6 visits. Only 3.4% of patients had an emergency department visit for back pain, and 1.1% were hospitalized for back pain in the past year.

Patient satisfaction was moderate overall, with a mean Likert score of 3.7 ± 1.0. Forty-eight percent of patients were satisfied or very satisfied with their treatment, 26.4% were neutral, and 25.6% were dissatisfied or very dissatisfied. On the Global Perceived Effect scale, 48.3% of patients reported feeling “much better” or “very much better” compared to before treatment, 26.1% reported feeling “slightly better,” 17.6% reported feeling “unchanged,” and 8.0% reported feeling “worse” or “very much worse.”

Comparison of secondary outcomes between the unimodal and multidisciplinary groups revealed significant differences in patient-reported and healthcare-utilization measures that were amenable to intervention, but not in low-frequency events. Patients in the multidisciplinary group used analgesic medications for significantly fewer days per month (5.2 ± 4.0 vs. 9.1 ± 4.2, *p* < 0.001), had fewer outpatient visits (2.0 ± 1.2 vs. 3.8 ± 1.9, *p* < 0.001), and reported higher patient satisfaction (mean Likert score: 4.1 ± 1.1 vs. 3.5 ± 0.9, *p* < 0.001) compared with patients in the unimodal group. However, emergency-department visits (3.4% overall) and hospitalizations for back pain (1.1% overall) were infrequent and did not differ significantly between groups (both *p* > 0.20). Additionally, 61.6% of patients in the multidisciplinary group rated their global improvement as “much improved” or “very much improved” on the GPE at 5 years, compared with 38.9% in the unimodal group (*p* < 0.001).

### 3.6. Sensitivity Analyses

Sensitivity analyses using complete-case analysis (excluding the 68 patients lost to follow-up) yielded results that were nearly identical to the primary analysis. The adjusted mean difference in ODI between the multidisciplinary and unimodal groups at 5 years was −7.9 points (95% CI: −10.7 to −5.1, *p* < 0.001), and the HR for surgical conversion associated with multidisciplinary care was 0.55 (95% CI: 0.35–0.86, *p* = 0.009). To address potential residual confounding by indication, we additionally estimated treatment effects using propensity-score (PS) methods. The PS was derived from a logistic model including age, sex, BMI, duration of pain, baseline NPRS and ODI, leg pain, smoking, TSK-11, PCS, and CCI. Inverse-probability-of-treatment weighting (IPTW) achieved good balance (standardized mean differences <0.10 for all covariates after weighting). IPTW-weighted estimates closely matched the primary multivariable results: 5-year adjusted ODI difference −7.6 points (95% CI −10.3 to −4.9, *p* < 0.001), NPRS difference −1.3 points (−1.8 to −0.8, *p* < 0.001), and surgical-conversion HR 0.61 (0.40–0.93, *p* = 0.02). PS-matched analysis (1:1 nearest-neighbor, caliper 0.2 SD of the logit PS, n = 208) yielded concordant estimates (HR 0.59, 95% CI 0.37–0.94). Additional sensitivity analyses using different model specifications produced consistent results.

## 4. Discussion

This 5-year retrospective cohort study extends the evidence base for non-surgical management of non-specific chronic low back pain (CLBP) by describing long-term real-world outcomes in a tertiary spine center in Northwest China. Three findings are clinically important. First, pain and disability improved most during the first year, but thereafter pain showed partial recurrence while function continued to improve, supporting the view that CLBP is a persistent, fluctuating condition rather than a self-limited episode and that pain and disability trajectories can diverge over time. Second, receipt of multidisciplinary biopsychosocial care was associated with substantially better 5-year pain, disability, and patient-reported outcomes than unimodal therapy, with the largest differences in patients with high psychosocial risk. Third, pain catastrophizing, leg pain, baseline disability, and smoking independently predicted surgical conversion, whereas multidisciplinary care was associated with a lower surgical rate.

### 4.1. Long-Term Trajectory of CLBP Outcomes

The observed pattern of rapid early improvement followed by partial recurrence of pain but sustained functional gain is consistent with trajectory research showing that low back pain often follows heterogeneous and recurrent courses rather than a single recovery pathway [21,22]. Prospective cohort evidence also indicates that a substantial proportion of patients continue to report pain after the acute phase, challenging the assumption that most non-specific low back pain resolves completely without ongoing management [23]. Importantly, the divergence between pain and disability trajectories in our cohort—with ODI continuing to improve between 12 months and 5 years, while NPRS rose slightly—suggests that patients can maintain or regain functional capacity even when some pain persists, consistent with the goals of contemporary pain rehabilitation that emphasize function and self-management rather than complete pain elimination.

These findings have practical implications for long-term care design. Health systems often remain organized around episodic visits, medication refills, imaging, and procedural escalation, even though guideline-concordant CLBP care requires sustained access to active physical and psychological interventions [24,25]. International guideline overviews consistently recommend reassurance, advice to remain active, exercise, and non-pharmacologic care as central management strategies for non-specific low back pain [26]. Exercise approaches such as motor-control training can reduce pain and disability, although no single exercise type is clearly superior for all patients [27]. Maintenance-oriented exercise and education may also reduce recurrence risk, which supports the need for booster sessions, home-based progression, and relapse-prevention planning after the initial treatment phase [28]. Digital and self-management programs may be useful adjuncts for sustaining engagement between clinic visits, particularly in large catchment areas where frequent in-person visits are difficult [29,30].

### 4.2. Multidisciplinary Biopsychosocial Care and Long-Term Outcomes

The observed advantage associated with multidisciplinary care in this cohort is clinically plausible. Earlier systematic reviews found that intensive multidisciplinary biopsychosocial rehabilitation with functional restoration improves pain and function in disabling CLBP [31]. Randomized evidence also suggests that active rehabilitation programs incorporating physical and cognitive-behavioral components can improve functional limitations compared with waiting-list control conditions [32]. A recent systematic review and meta-analysis of four randomized controlled trials (PEDro 5–8) found that combining exercise therapy with health education produced greater reductions in pain intensity (SMD −0.75, 95% CI −1.41 to −0.08, *p* = 0.03) and disability (SMD −0.24, 95% CI −0.38 to −0.10, *p* = 0.001) than usual medical care, with additional benefits for kinesiophobia [33]. These pooled estimates support the rationale for integrating structured exercise with education—core components of the multidisciplinary program evaluated here—rather than delivering either component alone. Broader reviews of psychological therapies in chronic pain similarly support cognitive-behavioral and self-management approaches [34].

The mechanisms of benefit likely extend beyond strengthening or flexibility alone. Cognitive-behavioral therapies for chronic pain have shown efficacy across pain-related outcomes, particularly when they target maladaptive beliefs, avoidance, coping, and distress [35]. Updated Cochrane evidence similarly supports psychological therapies, especially cognitive-behavioral approaches, for reducing disability and distress in chronic pain populations [35]. Pain neuroscience education may further improve pain knowledge, reduce threat appraisal, and support reconceptualization of pain as a modifiable protective experience rather than a direct marker of tissue damage [36]. The development of “Explaining Pain” approaches has emphasized this shift from structural explanation to pain-system education, which may help reduce catastrophizing and fear-based behavior [37]. Contemporary cognitive functional therapy integrates these principles with individualized movement, lifestyle, and behavioral retraining, offering a model for tailoring care to the patient’s dominant drivers of pain and disability [38,39].

### 4.3. Predictors of Surgical Conversion

Pain catastrophizing emerged as the strongest independent predictor of surgical conversion in our analysis. This finding is important because the Pain Catastrophizing Scale was designed to capture rumination, magnification, and helplessness in response to pain [40]. These cognitive-affective responses can amplify fear, vigilance, avoidance, and disability through the fear-avoidance pathway [41]. Systematic review evidence has long identified psychological factors as predictors of chronicity and disability in low back pain [42], and 5-year primary-care cohort data show that baseline pain intensity, disability, and psychosocial factors help predict longer-term outcomes [43]. Psychological factors are therefore not secondary or “non-organic” issues; they are part of the causal and prognostic architecture of persistent pain [44].

The association between leg pain/sciatica and surgical conversion was also expected, because radicular symptoms may indicate nerve-root involvement and are more likely to trigger imaging, specialist referral, and consideration of decompressive procedures [45]. Current smoking was another independent predictor, consistent with meta-analytic evidence linking smoking with both incident and prevalent low back pain, particularly chronic and disabling presentations [46]. Importantly, psychosocial factors may also shape postoperative outcomes. In lumbar disc surgery cohorts, fear of movement, passive coping, and negative outcome expectations have predicted ongoing pain and disability after surgery [47]. Mediation research likewise suggests that disability is not a simple consequence of pain intensity; beliefs, emotional responses, and behavioral adaptations help translate pain into functional limitation [48]. These findings support routine psychosocial screening before escalation to invasive treatment and suggest that psychological optimization should be considered part of surgical decision-making rather than an optional add-on.

### 4.4. Clinical Implications

Several clinical implications follow from these results. First, patients should be counseled that CLBP commonly fluctuates over years, so treatment goals should include durable self-management, maintenance of activity, and early response to relapse rather than complete and permanent pain elimination. Second, multidisciplinary care should be prioritized for patients with severe disability, high catastrophizing, fear of movement, work disruption, or repeated healthcare use. Third, surgical conversion should be interpreted within a broader biopsychosocial context: structural findings and neurological status remain important, but high psychosocial risk may increase healthcare seeking, reduce confidence in conservative care, and worsen postoperative recovery. A practical pathway would combine baseline risk stratification, individualized exercise progression, pain education, cognitive-behavioral support where needed, smoking-cessation counseling, and planned follow-up or booster sessions after the initial treatment window.

### 4.5. Strengths and Limitations

The main strength of this study is its long-term real-world design, which captures patients and treatment patterns that are often underrepresented in explanatory randomized trials. The use of repeated measures over 5 years, adjustment for clinical and psychosocial covariates, and evaluation of surgical conversion strengthen the clinical relevance of the findings. Nevertheless, the study should be interpreted with caution. Because treatment was allocated non-randomly according to clinician judgment and patient preference, confounding by indication cannot be fully excluded. Although we addressed this through multivariable adjustment and propensity-score sensitivity analyses, unmeasured confounding (e.g., motivation, socioeconomic resources, care-seeking behavior) may remain. Observational studies are also vulnerable to selection bias, residual confounding, and information bias [49,50].

Additional limitations include the single-center setting, which may restrict generalizability to primary care, rural hospitals, or other health systems. Follow-up interviews in years 3–5 were conducted by telephone, and treatments received outside our institution were not fully captured. Adherence to long-term home exercise, behavioral strategies, and lifestyle changes was not measured in detail, limiting inference about which components sustained benefit. Finally, surgical conversion reflects clinical decision-making as well as disease progression; patient preference, surgeon recommendation, access to care, and insurance factors may all influence whether surgery occurs.

### 4.6. Future Directions

Future research should move beyond asking whether non-surgical management works in general and should instead identify which care components work best for which patients, at what intensity, and for how long. Multicenter prospective cohorts and pragmatic randomized trials in China are needed to validate these findings, improve external validity, and test scalable multidisciplinary pathways. Research should also evaluate maintenance strategies after the first year, including booster exercise sessions, digital monitoring, remote coaching, and relapse-prevention protocols. Finally, mechanistic studies should examine how reductions in catastrophizing, fear avoidance, sleep disturbance, and work-related stress mediate long-term improvements and reduce the likelihood of surgical conversion.

## 5. Conclusions

In conclusion, this 5-year retrospective cohort study of 420 patients with non-specific CLBP (352 with complete 5-year data) found that non-surgical management was associated with substantial 12-month improvements in pain and disability. Function continued to improve through 5 years, whereas pain showed partial recurrence after the first year, underscoring the chronic, fluctuating nature of CLBP. Early multidisciplinary biopsychosocial care was associated with more favorable long-term functional outcomes, greater patient satisfaction, and a lower incidence of surgical conversion over 5 years, particularly in patients with high psychosocial risk. Because treatment allocation was not randomized, these are observational associations that do not prove causality; residual confounding by unmeasured factors cannot be excluded, and the consistency of the findings across multivariable, complete-case, Firth penalized, and propensity-score analyses supports the robustness of the observed associations but does not establish a causal effect of multidisciplinary care. These results support routine psychosocial screening and suggest, but do not establish, that early multidisciplinary referral may be associated with better long-term outcomes; they therefore warrant confirmation in pragmatic randomized trials.

## Figures and Tables

**Table 1:** Baseline demographic and clinical characteristics of the study cohort.

Characteristic	Total Cohort (n = 352)	Unimodal Group (n = 234)	Multidisciplinary Group (n = 118)	*p*-Value
Age, years (mean ± SD)	49.2 ± 11.8	50.1 ± 11.5	47.5 ± 12.1	0.04
Female, n (%)	199 (56.5)	132 (56.4)	67 (56.8)	0.95
BMI, kg/m^2^ (mean ± SD)	25.1 ± 3.8	25.2 ± 3.7	24.8 ± 4.0	0.35
Overweight (BMI 25–29.9), n (%)	114 (32.4)	78 (33.3)	36 (30.5)	0.60
Obese (BMI ≥30), n (%)	45 (12.8)	32 (13.7)	13 (11.0)	0.47
Duration of pain, months (median [IQR])	18 [6–48]	16 [5–45]	21 [8–52]	0.12
Duration of pain > 1 year, n (%)	231 (65.6)	148 (63.2)	83 (70.3)	0.19
Presence of leg pain, n (%)	168 (47.7)	109 (46.6)	59 (50.0)	0.55
Baseline NPRS (mean ± SD)	7.1 ± 1.4	7.0 ± 1.4	7.3 ± 1.3	0.06
Baseline ODI (mean ± SD)	44.8 ± 9.7	43.5 ± 9.2	47.4 ± 10.1	<0.001
TSK-11 Score (mean ± SD)	33.1 ± 6.5	31.8 ± 6.2	35.8 ± 6.4	<0.001
PCS Score (mean ± SD)	25.2 ± 7.9	23.5 ± 7.6	28.6 ± 7.8	<0.001
High PCS (>24), n (%)	184 (52.3)	102 (43.6)	82 (69.5)	<0.001
Current smoker, n (%)	95 (27.0)	65 (27.8)	30 (25.4)	0.65
Charlson Comorbidity Index (mean ± SD)	1.2 ± 1.5	1.3 ± 1.6	1.1 ± 1.4	0.28

Note: Values are presented as mean ± SD, median [IQR], or n (%). Abbreviations: BMI, body mass index; NPRS, Numerical Pain Rating Scale; ODI, Oswestry Disability Index; TSK-11, 11-item Tampa Scale for Kinesiophobia; PCS, Pain Catastrophizing Scale; CCI, Charlson Comorbidity Index; SD, standard deviation; IQR, interquartile range. *p*-values were derived from independent-samples *t*-tests (continuous variables) and chi-square tests (categorical variables).

**Table 2:** Univariable and multivariable Cox proportional hazards models for predictors of surgical conversion.

Predictor	Univariable HR (95% CI)	*p*-Value	Multivariable HR (95% CI)	*p*-Value
Age (per 10 years)	0.96 (0.87–1.06)	0.41	0.94 (0.85–1.04)	0.24
Female sex	1.08 (0.79–1.47)	0.63	1.12 (0.82–1.53)	0.47
BMI (per 5 kg/m^2^)	1.12 (0.91–1.38)	0.28	-	-
Duration of pain > 1 year	1.23 (0.85–1.78)	0.27	-	-
Presence of leg pain	2.21 (1.58–3.09)	<0.001	2.05 (1.45–2.89)	<0.001
Baseline NPRS (per 1 point)	1.15 (0.98–1.35)	0.08	-	-
Baseline ODI (per 1 point)	1.04 (1.02–1.05)	<0.001	1.03 (1.02–1.04)	<0.001
TSK-11 (per 10 points)	1.32 (0.98–1.78)	0.07	-	-
High PCS (>24)	3.42 (2.03–5.76)	<0.001	3.10 (1.80–5.34)	<0.001
Current smoker	1.68 (1.21–2.33)	0.002	1.55 (1.10–2.18)	0.01
Charlson Comorbidity Index	1.05 (0.92–1.20)	0.46	-	-
Multidisciplinary care (vs. unimodal)	0.47 (0.31–0.71)	<0.001	0.58 (0.38–0.88)	0.01

Note: Abbreviations: HR, hazard ratio; CI, confidence interval; NPRS, Numerical Pain Rating Scale; ODI, Oswestry Disability Index; TSK-11, 11-item Tampa Scale for Kinesiophobia; PCS, Pain Catastrophizing Scale; CCI, Charlson Comorbidity Index; BMI, body mass index. Dashes (-) indicate variables not retained in the multivariable model (*p* ≥ 0.20 in univariable analysis).

**Table 3:** Five-year secondary outcomes and patient-reported outcomes.

Outcome	Total Cohort (n = 352)	Unimodal Group (n = 234)	Multidisciplinary Group (n = 118)	*p*-Value
Analgesic use (days/month, mean ± SD)	7.8 ± 4.5	9.1 ± 4.2	5.2 ± 4.0	<0.001
Analgesic use ≥15 days/month, n (%)	79 (22.4)	65 (27.8)	14 (11.9)	<0.001
Outpatient visits (past year, mean ± SD)	3.2 ± 1.8	3.8 ± 1.9	2.0 ± 1.2	<0.001
Outpatient visits ≥6, n (%)	54 (15.3)	48 (20.5)	6 (5.1)	<0.001
Emergency department visits (past year), n (%)	12 (3.4)	10 (4.3)	2 (1.7)	0.21
Hospitalizations (past year), n (%)	4 (1.1)	4 (1.7)	0 (0.0)	0.31
Patient satisfaction (Likert 1–5, mean ± SD)	3.7 ± 1.0	3.5 ± 0.9	4.1 ± 1.1	<0.001
Satisfied/very satisfied, n (%)	169 (48.0)	97 (41.5)	72 (61.0)	<0.001
GPE: Much better/very much better, n (%)	170 (48.3)	98 (41.9)	72 (61.0)	<0.001
GPE: Unchanged/worse, n (%)	90 (25.6)	72 (30.8)	18 (15.3)	0.002

Note: Values are presented as mean ± SD or n (%). Abbreviations: GPE, Global Perceived Effect; SD, standard deviation. Patient satisfaction was rated on a 5-point Likert scale (1 = very dissatisfied to 5 = very satisfied); GPE was rated on a 7-point scale (1 = very much worse to 7 = very much better). Healthcare-utilization and medication-use data were collected by structured telephone interview at the 5-year follow-up and may be subject to recall bias.

## Data Availability

The raw data supporting the conclusions of this article will be made available by the authors on request.

## Abbreviations

The following abbreviations are used in this manuscript:

CLBP	Chronic low back pain
NPRS	Numerical Pain Rating Scale
ODI	Oswestry Disability Index
HR	Hazard Ratio
GBD	Global Burden of Disease
WHO	World Health Organization
ACP	American College of Physicians
STROBE	Strengthening the Reporting of Observational Studies in Epidemiology
IRB	Institutional Review Board
EMR	Electronic Medical Record
MRI	Magnetic Resonance Imaging
CT	Computed Tomography
NSAIDs	Non-steroidal anti-inflammatory drugs
TENS	Transcutaneous Electrical Nerve Stimulation
GPE	Global Perceived Effect
TSK-11	11-item Tampa Scale for Kinesiophobia
PCS	Pain Catastrophizing Scale
CCI	Charlson Comorbidity Index
BMI	Body Mass Index
IQR	Interquartile Range
SD	Standard Deviation
LMM	Linear Mixed-effects Model
CI	Confidence Interval
MICE	Multiple Imputation by Chained Equations
TLIF	Transforaminal Lumbar Interbody Fusion
